# PFOS Impairs Cognitive Function in Female Rats by Disrupting Astrocyte-Derived Estrogen–ERβ–NDRG2 Signaling Axis

**DOI:** 10.3390/toxics14070595

**Published:** 2026-07-06

**Authors:** Yue Su, Xiyang You, Zongqin Wang, Yufeng Tan, Jing Shao, Xiaohui Liu

**Affiliations:** Department of Environmental Health and Toxicology, School of Public Health, Dalian Medical University, Dalian 116044, China; 15603668146@163.com (Y.S.); youxiyang7056@163.com (X.Y.); wzq19990602@163.com (Z.W.); tanyufeng0914@163.com (Y.T.)

**Keywords:** perfluorooctane sulfonate, neurotoxicity, astrocyte, E2-ERβ signaling

## Abstract

Epidemiological investigations have indicated that females are particularly susceptible to perfluorooctane sulfonate (PFOS)-induced cognitive impairment, yet the mechanisms underlying this sex-specific vulnerability remain obscure. Estrogen and estrogen receptor β (ERβ) signaling are essential for female brain function, but their role in PFOS-induced neurotoxicity has not been explored. We therefore hypothesized that disruption of astrocyte-derived estrogen–ERβ signaling, leading to downregulation of N-myc downstream-regulated gene 2 (NDRG2) and subsequent synaptic dysfunction, contributes to PFOS-induced neurotoxicity in females. Female rats were exposed to PFOS for 30 days, followed by behavioral tests and hippocampal analysis. PC12 cells were treated with astrocyte-conditioned medium (ACM) to assess synaptic injury. Molecular docking was further performed to predict the binding affinity between PFOS and ERβ. In vivo, PFOS exposure impaired cognitive performance and caused hippocampal dysfunction, accompanied by decreased levels of estradiol (E2), aromatase (AROM), ERβ, N-myc downstream regulated gene 2 (NDRG2), and AMPA receptors (AMPARs), together with increased glial fibrillary acidic protein (GFAP) and Ca^2+^/calmodulin-dependent protein kinase II (CaMKII) in the hippocampus. In vitro, PFOS-exposed C6 cells showed reduced E2, AROM, ERβ, and NDRG2, along with elevated GFAP and extracellular glutamate concentration. PC12 cells treated with PFOS-ACM exhibited decreased synaptophysin (SYP), postsynaptic density protein 95 (PSD-95), and AMPARs, as well as increased CaMKII, indicative of synaptic injury. Pretreatment with E2 or the ERβ agonist diarylpropionitrile (DPN) could reverse these molecular alterations and mitigate neuronal dysfunction. Molecular docking revealed a strong binding affinity between PFOS and ERβ. Collectively, these findings support our hypothesis that PFOS impairs cognitive function in female rats by disrupting astrocyte-derived estrogen–ERβ–NDRG2 signaling, with NDRG2 as a potential downstream effector. This provides a mechanistic basis for the heightened female susceptibility to PFOS neurotoxicity and highlighting ERβ as a potential therapeutic target.

## 1. Introduction

Poly- and perfluoroalkyl substances (PFASs) are a class of synthetic chemicals used in commercial, agricultural, and industrial products. Perfluorooctane sulfonate (PFOS) is an end-product of PFASs, mainly as an additive for over 50 years. Due to its numerous applications, PFOS has been detected in various environmental matrices and biological samples globally, including the serum of polar animals, human breast milk, and placenta [1]. PFOS is highly resistant to natural degradation and therefore persists in the environment for decades, despite restrictions on its production and use in many countries [2]. A growing number of studies have shown that PFOS causes hepatic, endocrine and immunological dysfunction, as well as developmental effects [3]. Hence, concerns about PFOS increase due to its persistence in the environment and potential health effects.

Epidemiology investigations have revealed that early-life exposure to PFOS may be associated with neurodevelopmental deficits [4,5,6]. More recent evidence further suggests a potential link between PFOS exposure and an increased risk of neurodegenerative diseases, such as Alzheimer’s disease and Parkinson’s disease [7,8,9]. Despite these observations, consistent evidence on the relationship between PFOS exposure and neurodevelopmental outcomes in humans remains limited [10], likely due to the small sizes [11] and differences in developmental stages [12,13]. Therefore, animal studies are still necessary in evaluating the neurodevelopmental effects caused by PFOS. For example, a recent study reported that the negative association between cord serum PFOS levels and brain-derived neurotrophic factor (BDNF) was significant in female neonates but not in males, suggesting that females may be more vulnerable to PFOS neurotoxicity [14]. Previous studies have also shown that positive associations between prenatal PFASs exposure and neurodevelopmental outcomes are found in girls but not in boys [6,15]. These findings suggested that females were more susceptible to the neurotoxic effects induced by PFOS. Therefore, it was necessary to explore the mechanism and the potential molecular target for PFOS-induced neurotoxicity in females.

Estrogen plays a dominant role in female brain health, supporting synaptic plasticity, neuroprotection, and cognitive function [16,17]. Notably, astrocyte-derived estrogen has been shown to exert beneficial effects on neuroprotection and synapse development via activation of estrogen receptors (ERs) [18]. However, because females rely more heavily on this estrogen-dependent protective machinery, they may be particularly vulnerable when such pathways are disrupted by endocrine-disrupting chemicals (EDCs) like PFOS. Accordingly, understanding whether PFOS interferes with astrocyte-derived estrogen-ERβ signaling is critical for elucidating the mechanisms underlying female-specific neurotoxicity. Although estrogen receptors (ERs) are found throughout the brain, the two main genes, ERα and ERβ, do not show the same expression patterns. In particular, in the hippocampus and cortex, ERα mRNA levels are much lower than ERβ mRNA levels. Because of this difference, ERβ is generally considered to have a stronger effect on cognitive function in females [19].

PFASs are recognized as endocrine-disrupting chemicals (EDCs) that interfere with hormone biosynthesis, metabolism, or action, thereby affecting homeostatic control and reproduction [20]. Specifically, PFOS has been shown to disrupt lipid metabolism, damage cellular membranes, and alter signaling pathways through partial metabolism by cytochrome P450 enzymes. Accumulating evidence links PFOS to systemic endocrine disruption, including interference with steroid hormone synthesis and thyroid function. In vitro studies have demonstrated that PFOS can directly modulate steroidogenesis in adrenocortical cells, altering the production of key sex hormones such as estradiol and testosterone [21]. The relationship between PFOS exposure and hormone disturbance was also reported in adults [22]. However, the role of estrogen and ERβ signaling in PFOS-induced neurotoxicity in females was not clear. Previous studies in our lab have demonstrated that PFOS causes neuronal injuries through factors released from astrocytes [23,24], indicating that astrocyte–neuron crosstalk is a critical determinant of neurotoxic outcomes. Here, we hypothesize that PFOS disrupts ERβ signaling, leading to downregulation of NDRG2 in astrocyte, which may mediate downstream effects. To test this hypothesis, we assessed multiple markers reflecting key aspects of PFOS neurotoxicity. Astrocyte activation was monitored by glial fibrillary acidic protein (GFAP, a well-established marker of reactive gliosis) expression [25]. Synaptic integrity was evaluated through Ca^2+^/calmodulin-dependent protein kinase II (CaMKII, a central mediator of synaptic plasticity), PSD-95 (a postsynaptic scaffolding protein), and synaptophysin (a presynaptic vesicle protein) [26,27,28]. Excitotoxicity was assessed by extracellular glutamate levels and AMPA receptor (AMPAR) expression [29,30]. Estrogen signaling was examined by measuring estradiol (E2), aromatase (AROM), and ERβ levels [31]. NDRG2 was included as a potential downstream effector of ERβ based on its estrogen-regulated expression in astrocytes [32]. To support this hypothesis, we evaluated the neurobehavioral abilities and biochemical molecules related to estrogen and ERβ signaling in female rats exposed to PFOS in vivo. Then, the underlying mechanisms were investigated in both C6 cells exposed to PFOS and PC12 cells exposed to PFOS–astrocyte conditional medium (ACM) in vitro. The present study provides novel mechanistic insights into PFOS-induced neurotoxicity in females by demonstrating that PFOS disrupts astrocyte-derived estrogen–ERβ–NDRG2 signaling, leading to cognitive impairment.

## 2. Materials and Methods

### 2.1. Animals and Cells

#### 2.1.1. Animals

A total of 40 female specific pathogen-free (SPF) Sprague-Dawley (SD) rats (postnatal days 25, weighing 60–80 g) were obtained from the Animal Center of Dalian Medical University (Dalian, China). All animals were housed in stainless steel cages under controlled environmental conditions (temperature 23 ± 2 °C, humidity 55 ± 5%, 12 h light/dark cycle) with free access to standard rodent chow and filtered water. Animals were allowed to acclimate for 7 days prior to the start of the experiment. All experimental procedures were approved by the Institutional Animal Care and Use Committee of Dalian Medical University (Ethics No. AEE18060) and conducted in accordance with the National Institutes of Health Guide for the Care and Use of Laboratory Animals.

#### 2.1.2. Cells

The rat glioma C6 cell line (a well-established model for studying astrocyte biology [33], kindly provided by the Department of Toxicology, Dalian Medical University, China), and the rat adrenal pheochromocytoma PC12 cell line (a widely used neuronal model [34], Shanghai iCell Bioscience Inc., (Shanghai, China) (No. iCell-r024)) were used in the present study. Both cell lines were authenticated by the supplier and tested negative for mycoplasma contamination. C6 cells were cultured in DMEM (GIBCO, Waltham, MA, USA) supplemented with 10% fetal bovine serum (FBS, GIBCO, Waltham, MA, USA), 100 U/mL penicillin, and 100 µg/mL streptomycin at 37 °C in a humidified atmosphere containing 5% CO_2_. PC12 cells were maintained in DMEM supplemented with 10% heat-inactivated horse serum (GIBCO, Waltham, MA, USA), 5% FBS, and 1% penicillin/streptomycin under the same culture conditions. Cells were passaged at 80–90% confluence using 0.25% trypsin-EDTA (GIBCO, Waltham, MA, USA).

### 2.2. Sex as a Biological Variable (SABV) Statement

This study was undertaken to examine PFOS-induced neurotoxicity in female rats, drawing on epidemiological evidence that indicates sex-specific susceptibility, with females showing greater vulnerability to PFOS-related cognitive impairment [14,15]. The mechanistic focus on astrocyte-derived estradiol and ERβ signaling—pathways intrinsically linked to female hormonal regulation—further justified the use of a female-only model. All experiments were conducted exclusively in female Sprague-Dawley rats and female-derived C6 astrocyte and PC12 neuronal cell lines. Data were analyzed and presented as female-specific outcomes. The findings provide mechanistic insights into the female-predominant neurotoxic effects of PFOS and highlight the importance of considering sex as a biological variable in environmental neurotoxicology research.

### 2.3. Experimental Designs and Administration of Drugs

#### 2.3.1. Animal Treatment and Experimental Design

Following a 7-day acclimatization period, the animals were randomly divided into four groups with ten rats in each group: control (0 mg/kg·dw), PFOS low-dose (0.5 mg/kg·dw), PFOS medium-dose (1 mg/kg·dw), and PFOS high-dose (3 mg/kg·dw) [35,36,37]. To achieve the desired doses, PFOS (purity 98%, Sigma-Aldrich, St. Louis, MO, USA) was first dissolved in 0.5% Tween-20 (Solarbio, Beijing, China) in deionized water to prepare a stock solution at 5 mg/mL, which was then diluted with deionized water containing 0.5% Tween-20 to final working concentrations of 0, 0.5, 1.0, and 3.0 mg/mL; the final Tween-20 concentration was maintained at 0.5% across all solutions to ensure uniform solubility and consistent vehicle conditions. Animals were administered the respective PFOS solutions or vehicle control (0.5% Tween-20 in deionized water) once daily by oral gavage at a fixed volume of 5 mL/kg body weight, with the gavage volume adjusted daily based on the most recent body weight to ensure accurate dose delivery. The exposure lasted for 30 consecutive days (postnatal days 33–62), and the administration scheme is summarized in Table S1. Body weight was recorded every 3 days throughout the 30-day exposure period using an electronic balance (accuracy 0.1 g), and the data were used to monitor general health and to adjust the gavage volume accordingly. The body weight data are presented in Table 1 and described in the Results Section. The daily water consumption was also recorded. Cognitive function was evaluated using the Morris water maze. After behavioral testing, animals were euthanized under chloral hydrate anesthesia, and the hippocampus was isolated for biochemical analyses.

#### 2.3.2. Experimental Design for Cell Culture

For in vitro experiments, C6 astrocytes were seeded in 6-well plates at a density of 5 × 10^5^ cells/well and cultured in DMEM supplemented with 10% fetal bovine serum, 100 U/mL penicillin, and 100 µg/mL streptomycin at 37 °C in a humidified atmosphere containing 5% CO_2_ until 80–90% confluence. The cells were then treated with 10 nM 17β-estradiol (E2, Beyotime, Shanghai, China) or 10 nM DPN (diarylpropionitrile, >98%, Beyotime, Shanghai, China) (an ERβ-selective agonist), at concentrations selected based on previous studies [38,39]. The cells were divided into the following groups: control (serum-free medium with 0.05% DMSO, Solarbio, Beijing, China), E2 (exposed to 10 nM E2 for 24 h), PFOS (exposed to 50 µM PFOS for 24 h, according to the results in Figure S1), and E2 + PFOS (pretreated with E2 for 1 h, then exposed to PFOS for 24 h). After the respective treatments, the culture medium was removed, and the cells were washed twice with PBS to remove residual serum and treatment compounds. The cells were then incubated in serum-free DMEM (supplemented with 100 U/mL penicillin and 100 µg/mL streptomycin, without FBS) for 24 h. Following this incubation, the conditioned medium (ACM) from each group (control-ACM, E2-ACM, PFOS-ACM, and E2 + PFOS-ACM) was collected, centrifuged at 3000× *g* for 15 min at 4 °C to remove cell debris, and the supernatant was filtered through a 0.22 µm syringe filter (Millipore, Burlington, MA, USA) to eliminate any remaining cellular particles. The resulting ACM was either used immediately for neuronal treatment or aliquoted and stored at −80 °C for subsequent experiments. PC12 cells were seeded in 6-well plates at a density of 1 × 10^6^ cells/well and differentiated with 50 ng/mL nerve growth factor (NGF, Beyotime, Shanghai, China) for 48 h prior to treatment. The cells were then exposed to the respective ACM (control-ACM, E2-ACM, PFOS-ACM, or E2 + PFOS-ACM) for 24 h. After ACM treatment, PC12 cells were harvested for further analyses, including cell viability (MTT assay), Western blot, and immunofluorescence.

### 2.4. Morris Water Maze (MWM) Test

The MWM apparatus consisted of a circular pool (diameter 150 cm, height 50 cm) filled with water (23 ± 1 °C) rendered opaque by the addition of non-toxic white paint. The pool was divided into four quadrants (northeast, southeast, southwest, and northwest), and a removable escape platform (diameter 10 cm) was submerged 0.5 cm below the water surface in the center of the target quadrant (northeast), positioned approximately 15 cm from the pool wall. Visual cues (e.g., posters, lights) were placed around the room and remained constant throughout the experiment. During the acquisition phase (days 1–4), each rat received four training trials per day for four consecutive days, with an inter-trial interval of 15–20 min. In each trial, the rat was gently placed into the pool facing the wall from one of three starting positions (excluding the target quadrant) in a pseudo-randomized order, and was allowed to swim for a maximum of 90 s to locate the hidden platform. If the rat failed to find the platform within 90 s, it was gently guided to it and allowed to remain there for 5 s. Escape latency (time to reach the platform) was recorded for each trial using the EzVideo™ 5.70 Digital Video Tracking system (Accuscan Instruments, Inc., Columbus, OH, USA), and the daily average escape latency was calculated from the four trials and used for statistical analysis. On day 5 (probe trial), the platform was removed, and each rat was subjected to a single 60 s trial, released from the quadrant opposite to the target quadrant. The following parameters were recorded: (1) time spent in the target quadrant (where the platform had been located), and (2) the number of platform area crossings. All behavioral data were analyzed by an investigator blinded to the experimental groups.

### 2.5. Hematoxylin–Eosin (HE) Staining

The whole brain was fixed with 4% paraformaldehyde. For H&E staining, fixed brains were dehydrated through a graded ethanol series (70%, 80%, 90%, 95%, and 100%), cleared in xylene, and embedded in paraffin. Coronal hippocampal sections (5 µm) were cut using a rotary microtome (Leica RM2255, Leica Biosystems Nussloch GmbH, Nussloch, Germany). Sections were then deparaffinized in xylene, rehydrated through a graded ethanol series, and washed in distilled water. The sections were stained with Harris hematoxylin (Beyotime, Shanghai, China) for 5 min, followed by a 1–2 min rinse in running tap water for blueing. Subsequently, sections were differentiated in 0.3% acid alcohol for a few seconds, counterstained with alcoholic eosin for 2–3 min, dehydrated through a graded ethanol series, cleared in xylene, and mounted with neutral resin. Histological alterations in the hippocampal CA1, CA3, and DG regions were examined and imaged using a light microscope. Histological evaluation was performed by examining HE-stained sections for the following parameters: (1) neuronal arrangement and laminar organization; (2) nuclear morphology (presence of pyknotic nuclei); and (3) cell density. A semi-quantitative scoring system was not applied; the assessment was descriptive and focused on consistent morphological alterations observed across PFOS-treated animals.

### 2.6. Viability for C6 and PC12 Cells

Cell viability was assessed using the MTT assay (3-(4,5-dimethylthiazol-2-yl)-2,5-diphenyltetrazolium bromide, Solarbio, Beijing, China). After exposure, 100 µL MTT working solution was added into each hole in 96 wells. Following incubation at 5% CO_2_ and 37 °C for 2.5 h, 100 μL DMSO was added to dissolve the formazan blue formed by mitochondria reducing in the cell. The cell viability was determined by measuring the absorbency of the DMSO-dissolved solution at 595 nm with ELISA Reader (DG-I, Shanghai, China). The formula (absorbance value of exposure group − blank absorbance value)/(absorbance value of control group − blank absorbance value) × 100% was used to calculate the cell survival rate.

### 2.7. ELISA for E2 and Glu Detection

Estradiol (E2) concentration was measured because E2 is the primary estrogen in the brain and is critical for cognitive function [30]. According to the instructions, the concentrations of E2 was measured by ELISA kit supplied by Shanghai Lengton Bioscience Co., Ltd., Shanghai, China. Briefly, samples and standards were incubated in pre-coated wells for 60 min at 37 °C, followed by washing, addition of HRP-conjugated detection antibody, and TMB substrate development. The reaction was stopped with H_2_SO_4_, and absorbance was read at 450 nm using a microplate reader (Thermo 3550, Waltham, MA, USA).

Extracellular glutamate was quantified as an indicator of excitotoxicity, given its established role in PFOS-induced neuronal dysfunction [29]. Extracellular glutamate levels in astrocyte-conditioned medium were determined with a glutamate ELISA kit (Nanjing Jiancheng, China). Briefly, 0.5 mL of sample was mixed with reagents, and the absorbance at 340 nm was recorded before and after a 40 min incubation at 37 °C. The concentration was calculated from the absorbance change against a standard curve.

### 2.8. Western Blot

After protein concentration was determined using a BCA protein assay kit (Beyotime, Shanghai, China), equal amounts of protein (20 µg per lane) were loaded and separated by 10% SDS-PAGE. The separated proteins were then transferred onto polyvinylidene fluoride (PVDF) membranes (Millipore, Burlington, MA, USA). The membranes were blocked with 5% non-fat skim milk in Tris-buffered saline containing 0.1% Tween-20 (TBST) for 2 h at room temperature. After blocking, the membranes were incubated overnight at 4 °C with primary antibodies diluted in primary antibody dilution buffer. The following primary antibodies were used: rabbit polyclonal anti-rat ERβ (1:1000, Beyotime, Shanghai, China) and rabbit polyclonal anti-rat AROM (1:1000, Beyotime, Shanghai, China), mouse monoclonal anti-rat GFAP (1:1000, Beyotime, Shanghai, China), rabbit monoclonal anti-rat AMPAR (1:1000, Beyotime, Shanghai, China), rabbit monoclonal anti-rat CaMKII (1:1000, Beyotime, Shanghai, China), mouse monoclonal anti-rat NDRG2 (1:200, Santa Cruz, Dallas, TX, USA), and mouse monoclonal anti-β-actin (1:1000, ZSGB-BIO, Beijing, China). After three washes with TBST, the membranes were incubated with horseradish peroxidase (HRP)-conjugated secondary antibodies (Protein Tech, Wuhan, China): anti-rabbit IgG (1:4000) for ERβ, AROM, AMPAR, and CaMKII; and anti-mouse IgG (1:4000) for GFAP, NDRG2 and β-actin for 1 h at room temperature. Protein bands were visualized using an enhanced chemiluminescence (ECL) substrate (Beyotime, Shanghai, China), and chemiluminescent signals were captured using a ChemiDoc MP imaging system (Bio-Rad Laboratories, Hercules, CA, USA). Band intensities were quantified using ImageJ software (National Institutes of Health, Bethesda, MD, USA, version 1.4.3.67), and the gray values of target proteins were normalized to β-actin.

### 2.9. Immunofluorescence

PC12 cells grown on coverslips were fixed in 4% paraformaldehyde, permeabilized with 0.1% Triton X-100, and blocked with 5% BSA. The cells were then incubated overnight at 4 °C with primary antibodies against PSD-95 (1:200, AF1096, Beyotime, Shanghai, China) and synaptophysin (SYP, 1:200, WL03058, Wanleibio, Shenyang, China). After washing, the cells were incubated with FITC-conjugated goat anti-rabbit IgG (H + L) secondary antibody (1:200, WLA032a, Wanleibio, Shenyang, China) for 1 h at room temperature. Nuclei were counterstained with DAPI. Confocal images were acquired using a Nikon inverted fluorescence microscope. For quantification, the fluorescence threshold was uniformly adjusted, and mean fluorescence intensity was measured using ImageJ software (version 1.4.3.67). For each experimental group, the mean fluorescence intensity was normalized to the control group, and the results were expressed as percentage of the control value (%). Data were obtained from three independent experiments per group.

### 2.10. Molecular Docking for PFOS and ERβ

For molecular docking, the three-dimensional crystal structure of human estrogen receptor β (ERβ) was retrieved from the Protein Data Bank (PDB ID: 2J7X) and prepared using PyMOL (version 1.8.0, Schrödinger, LLC, New York, NY, USA) by removing water molecules and the co-crystallized ligand (4-hydroxytamoxifen), adding hydrogen atoms, and saving in PDB format. The three-dimensional structure of PFOS (CID: 74483) was obtained from the PubChem database (https://pubchem.ncbi.nlm.nih.gov, accessed on 16 May 2025) and energy-minimized using the MMFF94 force field to achieve a stable conformation, then saved in mol2 format. Molecular docking was performed using the CB-Dock2 web server (https://cadd.labshare.cn/cb-dock2/, accessed on 16 May 2025), which employs the AutoDock Vina (version 1.2.0) docking engine, with the binding site defined based on the co-crystallized ligand position and the grid box dimensions set to 20 × 20 × 20 Å^3^ to encompass the entire ligand-binding pocket. Docking was performed with an exhaustiveness value of 8, and a maximum of 5 docking poses were generated for each run. The docking poses were ranked based on their predicted binding free energy (ΔG, kcal/mol), and the pose with the lowest binding energy was selected as the most favorable binding conformation and visualized using PyMOL to illustrate the interactions between PFOS and the surrounding amino acid residues of ERβ.

### 2.11. Statistical Analysis

All experiments were conducted three times with at least three technical repeats each time. The results were expressed as mean ± standard deviation (SD), and the statistical analysis was performed via one-way analysis of variance (ANOVA), followed by the LSD and Dunnett’s T3 test using the SPSS 13.0 (SPSS Inc., Chicago, IL, USA). Differences were considered statistically significant at *p <* 0.05.

## 3. Results

### 3.1. PFOS Exposure Impairs Cognitive Function and Hippocampal Integrity in Female Rats

**Figure 1:**
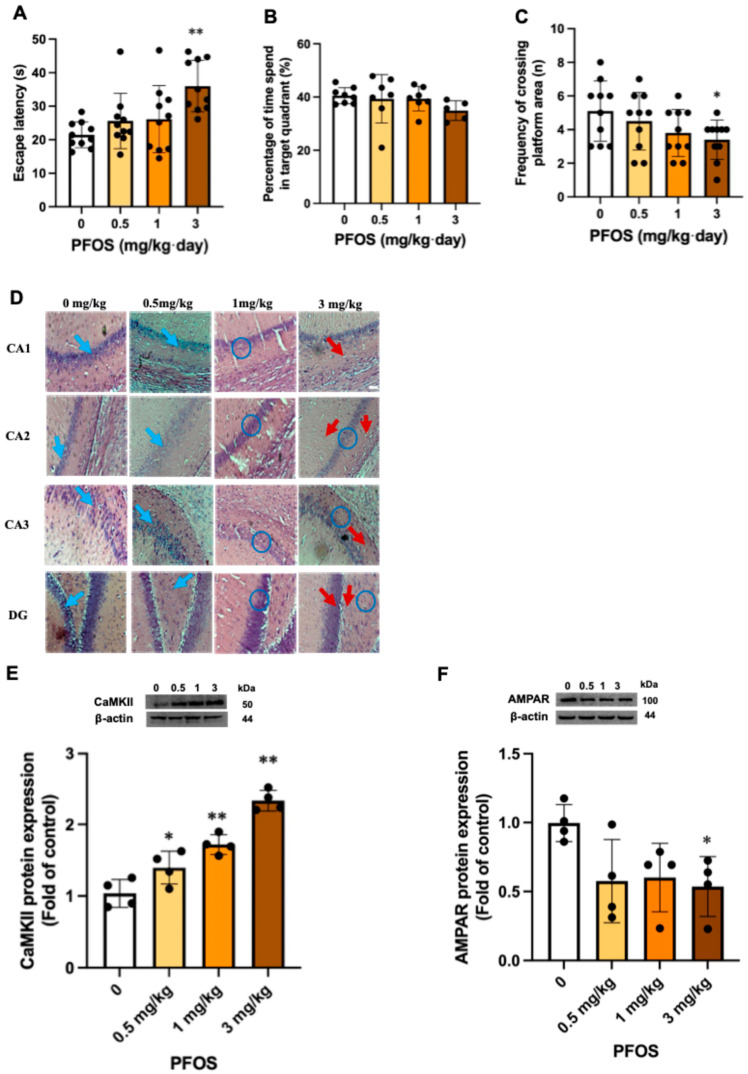
PFOS Exposure Impairs Cognitive Function and Hippocampal Integrity in Female Rats. (**A**) Escape latency result of Morris water maze (*n* = 10). (**B**) Percentage of time spent in target quadrant (*n* = 5–8). (**C**) Frequency of crossing platform (*n* = 10). (**D**) HE staining in hippocampus (400×, *n* = 3). Normal neurons are indicated by blue arrows; pyknotic nuclei by red arrows; areas of neuronal disorganization by blue circles. Scale bar in (CA1, 3 mg/kg) = 50 µm; applies to all panels. (**E**) CaMKII protein expression (*n* = 4). (**F**) AMPAR protein expression (*n* = 4). All data are presented as mean  ±  SD, * *p* < 0.05, ** *p* < 0.01, compared with the control group.

PFOS exposure induced dose- and time-dependent alterations in body weight gain. At the 1 mg/kg dose, body weight gain was significantly increased on days 6 and 15 compared with the control group. At the 3 mg/kg dose, body weight gain showed numerical decreases on days 3 and 30, but these differences did not reach statistical significance (Table 1). These observations suggest that PFOS may affect growth parameters in a complex, non-monotonic manner, although the biological significance of these changes remains to be determined. To assess the impact of PFOS exposure on learning and memory in female rats, we measured escape latency during acquisition trials, as well as the number of platform area crossings and the time spent in the target quadrant during probe trials. The results showed that PFOS exposure significantly increased escape latency (Figure 1A, *p* < 0.05). For the probe trial parameters, platform crossings showed a numerical decline (Figure 1B), and time spent in the target quadrant showed a decreasing trend (Figure 1C); however, these differences did not reach statistical significance (*p* > 0.05). Taken together, the significant prolongation of escape latency, together with the consistent but non-significant trends in probe trial parameters, suggests a PFOS-induced impairment in spatial learning in female rats. HE staining [40,41] was performed to assess morphological changes in the hippocampus of PFOS-exposed rats. In the control group, hippocampal neurons in the CA1, CA2, CA3, and DG regions exhibited densely packed and regularly arranged cell layers with clear nuclear morphology (blue arrow, Figure 1D). In contrast, PFOS-treated rats consistently displayed histological alterations across all four subfields, characterized by neuronal disorganization, loss of regular cell alignment (blue circle), and the presence of pyknotic nuclei (red arrow). These changes were dose-dependent and most evident in the 3 mg/kg PFOS group. CaMKII and AMPAR are involved in learning and memory processes [42]. As PFOS concentration increased, the protein expression of CaMKII (Figure 1E) was significantly upregulated, while the protein expression of AMPAR (Figure 1F) was significantly downregulated. The effects on body weight, neurobehavioral ability, histological alterations, and expressions of postsynaptic proteins indicated that PFOS caused developmental dysfunction in female rats, mainly affecting neurodevelopment.

### 3.2. PFOS Disrupts Astrocyte-Derived Estrogen–ERβ–NDRG2 Signaling in the Hippocampus

**Figure 2:**
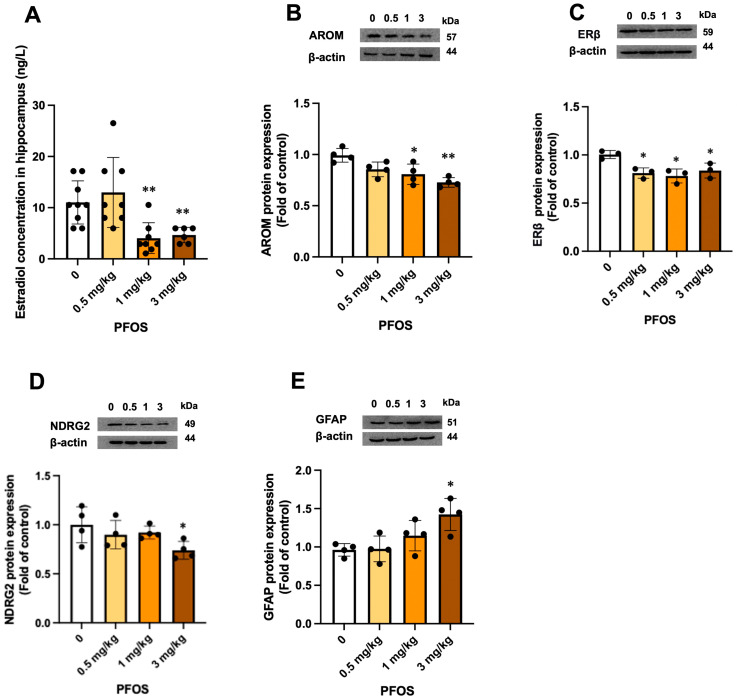
PFOS Disrupts Astrocyte-Derived Estrogen–ERβ–NDRG2 Signaling in the Hippocampus. (**A**) Brain estrogen (*n* = 6–9). (**B**) AROM protein expression (*n* = 4). (**C**) ERβ protein expression (*n* = 4). (**D**) NDRG2 protein expression (*n* = 4). (**E**) GFAP expression (*n* = 4). All data are presented as mean  ±  SD, * *p* < 0.05, ** *p* < 0.01, compared with the control group.

Extensive studies demonstrated that brain-derived E2 played important physiological and pathological roles [31]. To further investigate how PFOS causes injuries in neurodevelopment, we examined brain-derived E2. Compared with the control group, hippocampal E2 levels were significantly decreased at 1 mg/kg (4.0 ± 2.8 ng/L, *p* < 0.01) and 3 mg/kg (4.7 ± 1.4 ng/L, *p* < 0.01). In contrast, the 0.5 mg/kg dose (13.0 ± 6.4 ng/L) showed a non-significant increasing trend compared with the control (11.0 ± 4.0 ng/L, *p* > 0.05; Figure 2A). The expression of AROM (Figure 2B), which catalyses the conversion of androgens to estrogens in the brain, was also reduced significantly (control: 100.0 ± 5.8%; 0.5 mg/kg: 85.5 ± 6.1%; 1 mg/kg: 80.8 ± 8.6%, *p* < 0.05; 3 mg/kg: 72.8 ± 4.0%, *p* < 0.01). The results indicated disrupted regulation in E2 due to abnormal AROM caused by PFOS. In the present study, ERβ expression was decreased in the hippocampus of rats exposed to PFOS (control: 100.0 ± 3.4%; 0.5 mg/kg: 81.2 ± 4.3%, *p* < 0.05; 1 mg/kg: 78.2 ± 5.9%, *p* < 0.05; 3 mg/kg: 83.8 ± 6.3%, *p* < 0.05, Figure 2C). NDRG2, a recently identified gene, was regulated by E2 [43]. Compared with the control group, the expression of the NDRG2 protein (Figure 2D) in female rats was significantly decreased (control: 100.0 ± 15.9%; 0.5 mg/kg: 89.8 ± 12.5%; 1 mg/kg: 92.1 ± 5.7%; 3 mg/kg: 73.9 ± 7.8%, *p* < 0.05). The results showed that GFAP (Figure 2E) expression was significantly increased (control: 100.0 ± 7%; 0.5 mg/kg: 101.6 ± 14.4%; 1 mg/kg: 119.6 ± 17.1%; 3 mg/kg: 148.3 ± 18.1%, *p* < 0.05), indicating that astrocytes were in an active state. Therefore, these findings suggest that PFOS-induced neurodevelopmental injuries are associated with disruptions in astrocyte-derived E2 and NDRG2 levels in female rats.

### 3.3. E2 or ERβ Agonist Rescues PFOS-Induced Molecular Alterations in C6 Astrocytes

**Figure 3:**
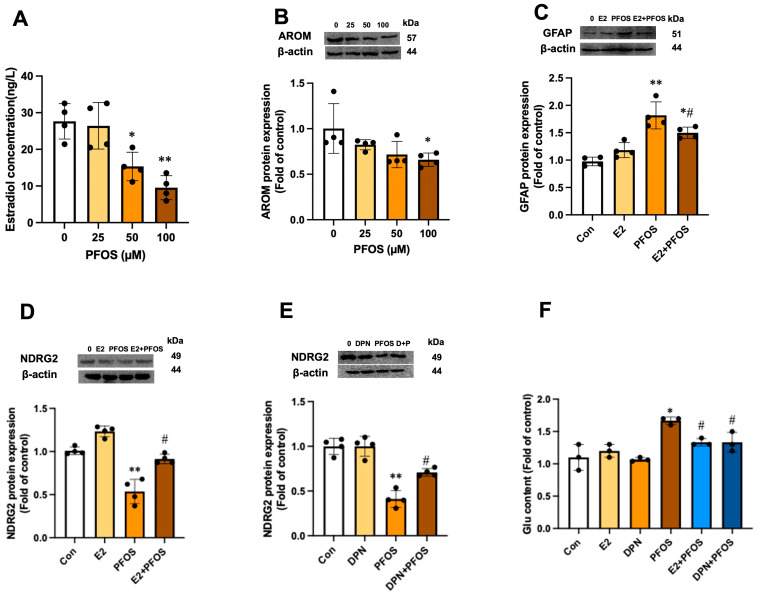
E2 or ERβ Agonist Rescues PFOS-Induced Molecular Alterations in C6 Astrocytes. (**A**) Estradiol concentration in C6 cells (*n* = 4). (**B**) AROM protein expression in C6 cells (*n* = 4). (**C**) GFAP expression in C6 cells (*n* = 4). (**D**) NDRG2 protein expression in C6 cells with E2 pretreatment (*n* = 4). (**E**) NDRG2 protein expression in C6 cells with DPN pretreatment (*n* = 4). (**F**) Glu content in C6 cells (*n* = 4). All data are presented as mean  ±  SD, * *p* < 0.05, ** *p* < 0.01, compared with control group; ^#^ *p* < 0.05, compared with the group pretreated with PFOS alone.

To determine whether PFOS directly affects astrocytes and whether ERβ activation is protective, we exposed C6 astrocytes to PFOS (50 µM) with or without E2 or DPN pretreatment. Cell viability was significantly reduced by PFOS exposure (Figure S1; control: 100.0 ± 4.8%; PFOS: 79.8 ± 3.0%, *p* < 0.05). E2 pretreatment partially restored viability (90.5 ± 1.7%, *p* < 0.05 vs. PFOS), and DPN pretreatment showed a similar protective effect (88.3 ± 0.8%, *p* < 0.05 vs. PFOS). PFOS exposure significantly decreased the E2 concentration in the culture medium (Figure 3A; control: 27.6 ± 4.2 ng/L; 25 µM: 26.4 ± 5.5 ng/L; 50 µM: 15.3 ± 3.4 ng/L, *p* < 0.05; 100 µM: 9.5 ± 2.8 ng/L, *p* < 0.01) and AROM expression (Figure 3B; control: 100.0 ± 12.5%; 25 µM: 82.4 ± 4.7%; 50 µM: 71.7 ± 12.5%; 100 µM: 66.0 ± 6.3%, *p* < 0.05). In addition, PFOS (50 µM) significantly increased GFAP expression (Figure 3C; control: 100.0 ± 6.8%; PFOS: 181.8 ± 21.4%, *p* < 0.01), indicating astrocyte activation. Importantly, PFOS (50 µM)-induced downregulation of NDRG2 was significantly alleviated by E2 pretreatment (Figure 3D; PFOS: 59.2 ± 8.5%; E2 + PFOS: 88.9 ± 2.4%, *p* < 0.05 vs. PFOS). Similarly, pretreatment with the ERβ-selective agonist DPN effectively rescued NDRG2 expression (Figure 3E; PFOS: 45.7 ± 9.5%; DPN + PFOS: 70.8 ± 3.7%, *p* < 0.05 vs. PFOS).

Consistent with previous studies showing that PFOS disrupts the glutamate–glutamine cycle [23,44], extracellular glutamate levels were significantly increased in PFOS-ACM (Figure 3F; control-ACM: 100.0 ± 8.2%; PFOS-ACM: 166.7 ± 4.7%, *p* < 0.05). This increase was significantly mitigated by E2 (133.3 ± 4.7%, *p* < 0.05 vs. PFOS) and DPN (133.3 ± 2.5%, *p* < 0.05 vs. PFOS) pretreatment. Together, these results demonstrate that PFOS directly damages C6 astrocytes by reducing E2 levels and disrupting ERβ-NDRG2 signaling, and that activation of ERβ with E2 or DPN effectively rescues these alterations.

### 3.4. E2 or DPN Pretreatment in Astrocytes Protects Neurons from PFOS-Induced Synaptic Dysfunction

**Figure 4:**
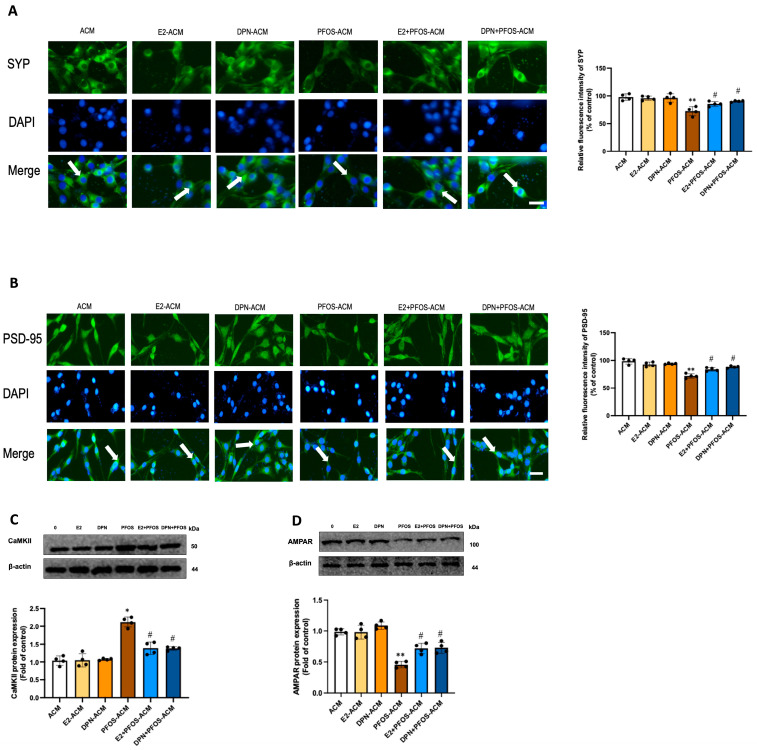
E2 or DPN Pretreatment in Astrocytes Protects Neurons from PFOS-Induced Synaptic Dysfunction. (**A**) Immunofluorescence staining for SYP in PC12 cells (*n* = 4, 400×, scale bar = 20 µm). White arrows indicate representative SYP-positive puncta. (**B**) Immunofluorescence staining for PSD-95 in PC12 cells (*n* = 4, 400×, scale bar = 20 µm). White arrows indicate representative PSD-95-positive puncta. (**C**) CaMKII protein expression in PC12 cells (*n* = 4). (**D**) AMPAR protein expression in PC12 cells (*n* = 4). All data are presented as mean  ±  SD, * *p* < 0.05, ** *p* < 0.01, compared with control group; ^#^ *p* < 0.05, compared with the group pretreated with PFOS alone.

To investigate whether astrocyte-derived E2-NDRG2, disrupted by PFOS, influences neuron and synapse development in PC12 cells, the conditional medium was collected from C6 cells exposed to PFOS. The viability (Figure S2) in PC12 cells treated with PFOS-ACM was notably reduced, but could be improved with PFOS-ACM pretreated with E2 or DPN. Subsequently, markers for presynaptic and postsynaptic membranes—synaptophysin (SYP), postsynaptic density protein 95 (PSD-95), CaMKII, and AMPAR—were examined in PC12 cells. Immunofluorescence analysis revealed that PFOS-ACM significantly decreased the fluorescence intensity of both SYP and PSD-95 compared with control-ACM. For SYP (Figure 4A), PFOS-ACM reduced fluorescence to 74.1% of control (*p* < 0.01), while E2 + PFOS-ACM (87.5% of control) and DPN + PFOS-ACM (92.5% of control) significantly attenuated this reduction (*p* < 0.05 vs. PFOS-ACM). Similarly, PSD-95 fluorescence (Figure 4B) was decreased by PFOS-ACM to 72.8% of control (*p* < 0.01), with partial restoration by E2 (84.8% of control) and DPN (89.5% of control).

Western blot analysis further confirmed that PFOS-ACM upregulated CaMKII expression (Figure 4C) and downregulated AMPAR expression (Figure 4D). PFOS-ACM increased CaMKII to 203.9% of control (*p* < 0.01), while E2 and DPN pretreatment reduced this increase to 133.0% and 132.5% of control, respectively (*p* < 0.05 vs. PFOS-ACM). Conversely, PFOS-ACM decreased AMPAR expression to 46.1% of control (*p* < 0.01), with partial restoration by E2 (72.5% of control) and DPN (73.5% of control). The findings from cell viability and synapse development assessments further supported that PFOS damaged neurons by impairing the protective function of astrocyte-derived E2-ERβ signaling, with NDRG2 expression being concurrently reduced.

### 3.5. Molecular Docking of Estrogen Receptor β with PFOS

**Figure 5:**
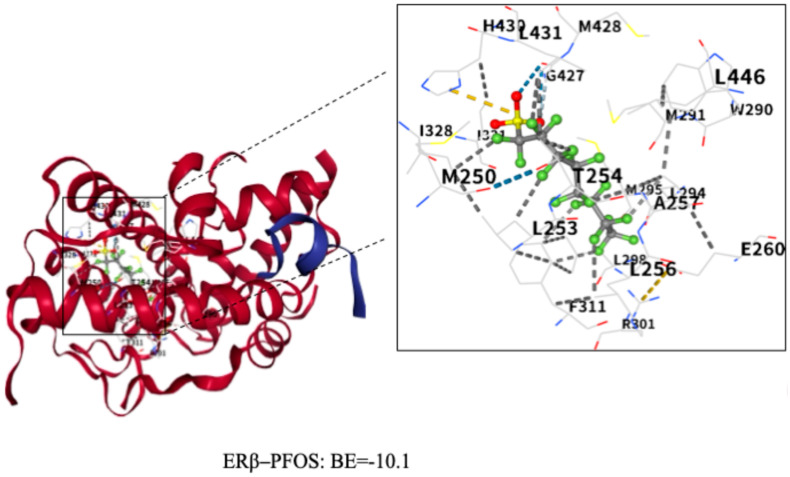
Molecular docking of estrogen receptor β (ERβ) with PFOS. The protein backbone is shown as a dark red cartoon representation, with a flexible loop region highlighted in dark blue. PFOS is depicted as a ball-and-stick model with CPK coloring: gray, carbon; white, hydrogen; red, oxygen; blue, nitrogen; yellow, sulfur; and green, fluorine. Intermolecular interactions are denoted by dashed lines: blue, hydrogen bonds; black, hydrophobic interactions; and yellow, π–π stacking. Key interacting amino acid residues are labeled with single-letter codes and sequence numbers. BE: binding energy.

To investigate whether PFOS can directly interact with ERβ, we performed molecular docking using the CB-Dock2 platform with the human ERβ crystal structure (PDB ID: 2J7X) and the PFOS structure from PubChem (CID: 74483). The docking simulation revealed a strong binding affinity between PFOS and ERβ (Figure 5), with a predicted binding free energy (ΔG) of −10.1 kcal/mol—substantially lower than the threshold for strong protein-ligand interactions (ΔG < −7.0 kcal/mol)—indicating that PFOS can spontaneously form a stable complex with ERβ. Analysis of the docking pose revealed that PFOS fits into the ligand-binding pocket of ERβ, the same pocket occupied by endogenous 17β-estradiol (E2), with interactions stabilized by hydrogen bonds (e.g., Glu305, Arg346) and hydrophobic contacts (e.g., Phe356, Leu384, Met336) [45]. This binding mode, characteristic of high-affinity ERβ ligands, suggests that PFOS may act as a competitive inhibitor of E2 binding [46]. This computational prediction is strongly supported by our experimental findings: PFOS exposure reduced ERβ expression in hippocampal tissue and C6 astrocytes, while the ERβ-selective agonist DPN rescued PFOS-induced NDRG2 downregulation, glutamate dysregulation, and synaptic dysfunction. Collectively, these data provide a structural rationale for PFOS-induced disruption of ERβ signaling, identifying competitive binding to ERβ as a potential initiating event in the cascade leading to cognitive impairment in female rats. 

## 4. Discussion

Accumulating evidence has shown that PFOS could disrupt brain function, especially in females [47,48]. However, the potential molecular mechanisms largely remain unknown. The present study demonstrates that PFOS impairs cognitive function in female rats through disruption of astrocyte-derived estrogen–ERβ–NDRG2 signaling. Specifically, our multi-level evidence (in vivo, in vitro, pharmacological rescue, and molecular docking) supports the following mechanistic cascade: PFOS enters the brain, where it suppresses astrocytic estradiol synthesis through aromatase inhibition and simultaneously binds directly to ERβ. Further in vitro investigation revealed that PFOS exposure in C6 astrocytic cells reduces E2 synthesis and disrupts ERβ signaling, which in turn impairs neuronal function. This dual disruption leads to downregulation of NDRG2, which in turn impairs glutamate uptake and subsequently triggers aberrant CaMKII activation and AMPA receptor dysfunction. Ultimately, these events result in synaptic dysfunction and cognitive deficits. Importantly, this pathway is at least partially reversible by ERβ activation, confirming its causal role in PFOS neurotoxicity.

### 4.1. PFOS Induces Systemic and Hippocampal-Dependent Deficits in Female Rats

Body weight gain was monitored as a general health indicator during the exposure period. PFOS exposure was associated with dose- and time-dependent alterations in body weight: an increase at the early stage of low-dose exposure and a decrease at later stages of high-dose exposure. Similar body weight changes have been reported in both human epidemiological studies and animal experiments following PFOS exposure [49,50]. While these observations are consistent with the metabolic-disrupting effects of PFOS reported in the literature [48], the biological significance and underlying mechanisms of the body weight changes observed in the present study remain unclear, given the non-monotonic and time-limited nature of the effects. Therefore, these findings should be interpreted with caution. In addition to changes in body weight, we also found that early PFOS exposure could induce learning and memory disability in female rats. PFOS could induce disordered brain function in animals in different life stages [36,51,52]. According to the time period when PFOS exposure (from postnatal day 33 to postnatal day 62) occurred in the present study, the results indicated that juveniles were more susceptible to PFOS exposure in the period, requiring more attention.

Having established the behavioral and structural deficits, we next examined synaptic protein alterations that may underlie these impairments. Histopathology changes and synapse proteins (CaMKII and AMPARs) alteration could be observed in the hippocampus of female rats exposed to PFOS in the present study. Experiments in PC12 cells also showed that PFOS-ACM could affect the expression of SYP and PSD-95, which also indicated the impairment of PFOS-ACM in the synapse development. Hippocampus is very important for learning and memory, and is maintained by both morphological and molecular characteristics [53]. Hence, the synaptic proteins CaMKII and AMPARs were evaluated in the present study. CaMKII is central to synaptic plasticity and could induce trafficking of AMPARs and other proteins to the synapse in a sequential fashion [54]. Hence, the disturbed arrangement, fracturing of cells, and altered synapse proteins in the hippocampus exposed to PFOS indicated a poor flow of information along DG-CA3-CA1, interrupted plasticity in connecting circuits, and damaged cognitive function.

### 4.2. PFOS Interferes with Astrocyte-Derived Estrogen-ERβ Signaling

To explore the upstream signals that may drive the observed synaptic and cognitive deficits, we investigated whether PFOS disrupts brain-derived estrogen signaling in the hippocampus. Endogenous hippocampal E2, not peripheral, could protect the brain [55], and is the key factor affecting the novel object recognition abilities of females [19,56]. Consistent with the report in epidemiological studies [22,57] and experiments with human placental syncytiotrophoblasts [58], we found a decreased hippocampal E2 concentration and AROM in the PFOS exposure group, both in vivo and in vitro. AROM is the key enzyme for the biosynthesis of estrogens [59]. Hence, the results disclosed that the decreased E2 concentration was attributed to the mediation of AROM by PFOS. Unlike the present result, an increased E2 level was observed in H295R cells exposed to PFOS [21]. The different effect of PFOS on E2 concentration might be associated with the difference in cell types [60], which needs further exploration. As mentioned, estrogen performs its function in the brain through estrogen receptors (ERα and ERβ) [61]. Different from ERα, ERβ is primarily expressed in non-gonadal tissues and is involved in cognitive activities through E2-ERβ signaling [62]. Notably, stimulation of hippocampal ERβ regulates synaptic plasticity and, in turn, improves performance on hippocampus-dependent memory tasks [63]. Among the downstream molecules of E2-ERβ signaling, a novel gene named NDRG2 has been shown to have a tissue-specific biological function [64]. The present study showed that NDRG2 expression was decreased in the hippocampus of young female rats exposed to PFOS. PFOS had estrogen-like activity, which could interact with human and rainbow trout ERs [65]. Hence, PFOS might replace estrogen to bind ERβ competitively, as shown in molecular docking analysis, resulting in decreased NDRG2 in the present study. The speculation was confirmed by the finding that C6 cells pretreated with DPN could enhance NDRG2 expression and reduce the following injuries in PC12 cells exposed to PFOS-ACM.

Having identified ERβ as an upstream regulator, we next examined the functional role of its downstream target NDRG2 in astrocytes. NDRG2 is widely expressed in the cytoplasm of astrocytes [66]. The decreased expression of NDRG2 and the increased GFAP in C6 cells further supported the regulation of PFOS on NDRG2 in activated astrocytes. NDRG2 could facilitate astroglial glutamate uptake to protect the brain from glutamate excitotoxicity through increasing the function of Na^+^/K^+^-ATPase β1 mediated by E2 and ERβ binding [67]. Consistent with the previous results [23,44], the present study also showed that the glutamate content was increased in PFOS-ACM, while E2 or DPN could mitigate the effect of PFOS-ACM on glutamate. The increased glutamate might be attributed to the inhibited effect of NDRG2 on Na^+^/K^+^-ATPase β1 through decreased E2-ERβ binding in C6 cells exposed to PFOS. Abnormal glutamate could open the channels for calcium, resulting in calcium overload in neurons exposed to PFOS [68], followed by increased CaMKII [69]. The disturbed expression of CaMKII, AMPAR, SYP, and PSD-95 in the hippocampus exposed to PFOS and in PC12 cells exposed to PFOS-ACM further implied that PFOS affected the glutamate uptake of astrocytes through the inhibitory effect of NDRG2 on Na^+^/K^+^-ATPase β1.

The marked increase in extracellular glutamate following PFOS exposure (Figure 3F) suggests that impaired glutamate uptake—likely due to NDRG2 downregulation—contributes to excitotoxic neuronal injury. This finding is consistent with the well-established role of glutamate dysregulation in PFOS neurotoxicity and further supports the protective function of ERβ-NDRG2 signaling in maintaining glutamate homeostasis. Importantly, the ability of E2 and DPN to mitigate glutamate accumulation reinforces the causal link between ERβ activation and the preservation of astrocytic glutamate handling.

### 4.3. Astrocytic ERβ-NDRG2 Signaling as a Rheostat Balancing Neuroprotection and Injury

Having established the molecular pathway from ERβ to NDRG2 and glutamate dysregulation, we now consider how this pathway fits into the broader context of astrocytic function and its dual role in neuroprotection and neurodegeneration. Reactive astrocytes may act as a double-edged sword in PFOS-induced neurotoxicity, exhibiting both deleterious and protective actions. On the one hand, PFOS directly injures astrocytes by disrupting the glutamate–glutamine cycle, downregulating GLT-1 expression, and enhancing inflammatory responses [23,24,70]. The release of pro-inflammatory mediators, such as TNF-α, together with the accumulation of extracellular glutamate, represents the detrimental facet of astrocytic activation. On the other hand, under the same toxic insult, astrocytes also mount compensatory protective responses, including the secretion of glial cell line-derived neurotrophic factor and the activation of CaMKII–DLG1 signaling [24]. This pattern of “protective injury” is not unique to PFOS; it reflects a broader paradigm of glial responses to diverse environmental toxicants, including heavy metals, pesticides, and other neurotoxic compounds [71]. Thus, elucidating the molecular switch that governs the balance between these opposing astrocytic states has emerged as a key research priority. In this context, astrocyte-derived estrogen–ERβ–NDRG2 signaling appears to play a central regulatory role. PFOS markedly perturbs astrocytic estrogen–ERβ signaling and reduces NDRG2 expression. Pretreatment with E2 or the ERβ-selective agonist DPN partially reverses the downregulation of NDRG2, restores glutamate homeostasis, and prevents synaptic dysfunction in neurons. Taken together, these findings suggest that astrocytic ERβ signaling, potentially via NDRG2, may act as a critical rheostat that biases the system toward neuroprotection. When this pathway is disrupted by PFOS—which exhibits high binding affinity for ERβ—the protective arm is attenuated, permitting injurious mechanisms to predominate and ultimately leading to cognitive deficits in exposed animals.

### 4.4. Limitations and Future Directions

Finally, several methodological and conceptual limitations of the present study should be acknowledged to guide interpretation and inform future investigations. While our in vitro experiments using the ERβ agonist DPN support the involvement of ERβ signaling in PFOS-induced neurotoxicity, direct genetic manipulation of NDRG2 (e.g., via knockdown or overexpression) was not performed. Therefore, the causal role of NDRG2 as an essential mediator remains to be definitively established. Furthermore, astrocyte-specific changes in hippocampal tissue were not directly demonstrated by cell-type-specific analysis (e.g., GFAP + ERβ co-immunostaining), as ERβ and NDRG2 reductions were measured in whole-tissue homogenates. In addition, systematic neuronal counting was not performed for histological assessment, which was primarily descriptive. Finally, the findings are derived from rodent models and cell lines, and whether the same mechanisms operate in humans remains to be determined. Future studies employing astrocyte-specific NDRG2 manipulation, immunohistochemical co-localization, and human-relevant models are warranted to validate these findings.

## 5. Conclusions

**Figure 6:**
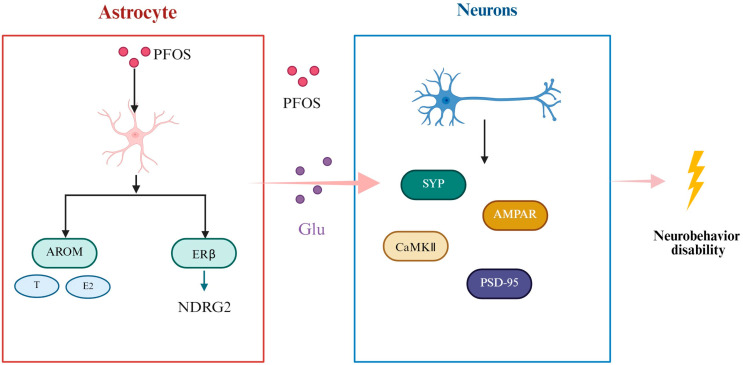
Mechanism hypothesis of astrocyte-derived estrogen in PFOS-induced neurobehavior disability. PFOS disturbed synapse function (abnormal expression of CaMKII, AMPARs, SYP and PSD-95) through astrocyte-derived E2-ERβ signaling disruption (inhibition of AROM for E2 and interacting with ERβ as E2 mimic).

Our findings suggested a novel mechanism underlying the female-specific susceptibility to PFOS neurotoxicity: disruption of astrocyte-derived E2-ERβ signaling, leading to downregulation of NDRG2 as a potential downstream effector (Figure 6). By demonstrating that an environmental EDC hijacks a sex-specific protective pathway, this study provides mechanistic insights into sex differences in environmental neurotoxicity and highlights ERβ as a potential therapeutic target for mitigating EDC-induced cognitive impairment in women. Nevertheless, we acknowledge that the causal role of NDRG2 as a downstream mediator remains to be further validated.

## Figures and Tables

**Table 1:** Increased body weigh in female rat (g, $\bar{x}$ ± *S*).

Day	0 mg/kg·dw	0.5 mg/kg·dw	1 mg/kg·dw	3 mg/kg·dw
3	18.2 ± 1.8	17.5 ± 2.6	19.4 ± 2.7	16.3 ± 2.6
6	34.0 ± 3.9	37.1 ± 4.2	39.3 ± 5.7 *	35.7 ± 4.7
9	56.7 ± 16.3	51.8 ± 4.4	54.2 ± 6.2	52.7 ± 8.4
12	66.5 ± 6.7	68.1 ± 4.8	72.9 ± 8.5	70.4 ± 9.1
15	80.7 ± 7.6	84.1 ± 6.4	89.0 ± 9.3 *	85.4 ± 10.5
18	93.5 ± 10.3	94.5 ± 9.4	101.7 ± 10.0	97.5 ± 11.0
21	103.6 ± 12.2	104.9 ± 11.7	111.8 ± 12.3	106.9 ± 10.7
24	113.8 ± 12.9	116.5 ± 12.4	123.2 ± 14.7	118.2 ± 12.3
27	125.3 ± 14.9	123.4 ± 13.3	134.2 ± 16.2	124.4 ± 12.7
30	131.6 ± 15.2	128.5 ± 15.8	141.4 ± 17.1	129.4 ± 13.8

Note: *: compared with the control, *p* < 0.05.

## Data Availability

The raw data supporting the conclusions of this article will be made available by the authors on request.

## Supplementary Materials

The following supporting information can be downloaded at https://www.mdpi.com/article/10.3390/toxics14070595/s1, Figure S1: Cell ability of C6 cells exposed to PFOS; Figure S2: Cell ability of PC12 cells exposed to PFOS-ACM; Figure S3: Experimental timeline of the in vivo study; Figure S4: Full-length, uncropped Western blot images. Original, unadjusted scans of the representative Western blot membranes for (A) Figure 1E-CaMKII and β-actin (B) Figure 3C-GFAP and β-actin; Figure S5: Full-length, uncropped Western blot images. Original, unadjusted scans of the representative Western blot membranes for Figure 4C-CaMKII and β-actin; Table S1: PFOS administration protocol.
