# Supplementary material for: PFOS Impairs Cognitive Function in Female Rats by Disrupting Astrocyte-Derived Estrogen–ERβ–NDRG2 Signaling Axis
Source: Toxics. 2026 Jul 6;14(7):595. doi: 10.3390/toxics14070595 (PMC13431314; doi:10.3390/toxics14070595)

Table S1: PFOS administration protocol

Group	Dose (mg/kg·dw)	Solution Concentration (mg/mL)	Gavage Volume (mL/kg)
Control	0	0	5 mL/kg
Low dose	0.5	0.5	5 mL/kg
Medium dose	1.0	1.0	5 mL/kg
High dose	3.0	3.0	5 mL/kg

All solutions were prepared in 0.5% Tween-20 in deionized water. Gavage volume was adjusted daily based on body weight.

Fig.S1

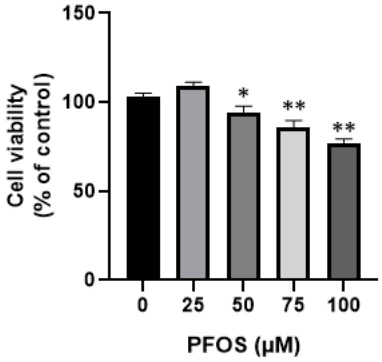

Fig.S1. Cell ability of C6 cells exposed to PFOS. All data are presented as mean ± SEM, $n = 3$ for each group. $^{*}P < 0.05$, $^{**}P < 0.01$, compared with control group.

Fig.S2

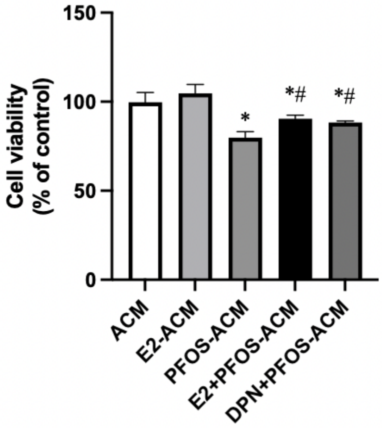

Fig.S2. Cell ability of PC12 cells exposed to PFOS-ACM. All data are presented as mean ± SEM, $n = 3$ for each group. $^{*}P < 0.05$, compared with ACM; $^{\#}P < 0.05$, compared with PFOS-ACM.

Fig.S3

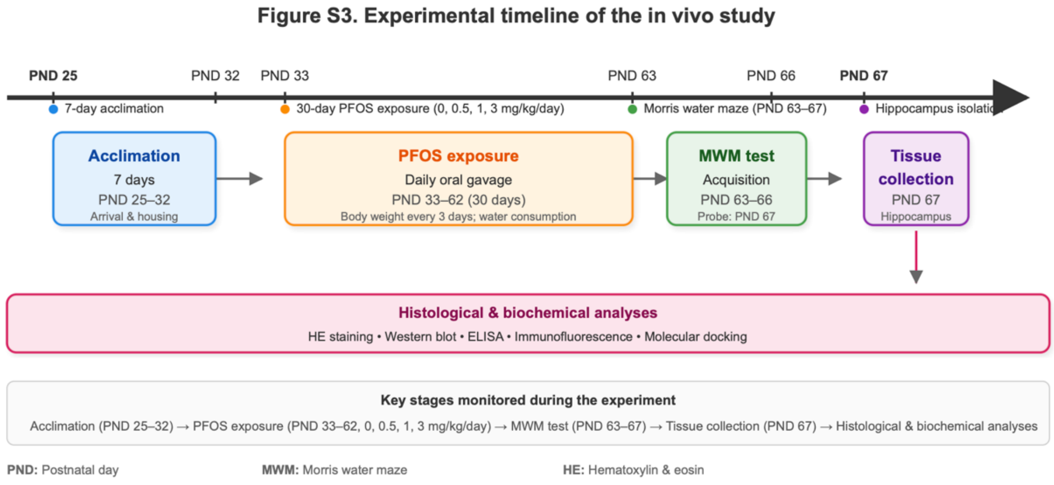

Experimental timeline of the in vivo study. Female SD rats (PND 25) were acclimated for 7 days and then exposed to PFOS (0, 0.5, 1, and 3 mg/kg/day) by oral gavage for 30 consecutive days (PND 33–62). Body weight was monitored every 3 days throughout the exposure period. The Morris water maze (MWM) test was conducted during PND 63–66 (acquisition phase), followed by a probe trial on PND 67. Immediately after the probe trial, hippocampal tissues were collected for histological and biochemical analyses, including HE staining, Western blot, ELISA, immunofluorescence, and molecular docking.

Fig.S4 for Fig.1E

CaMKII

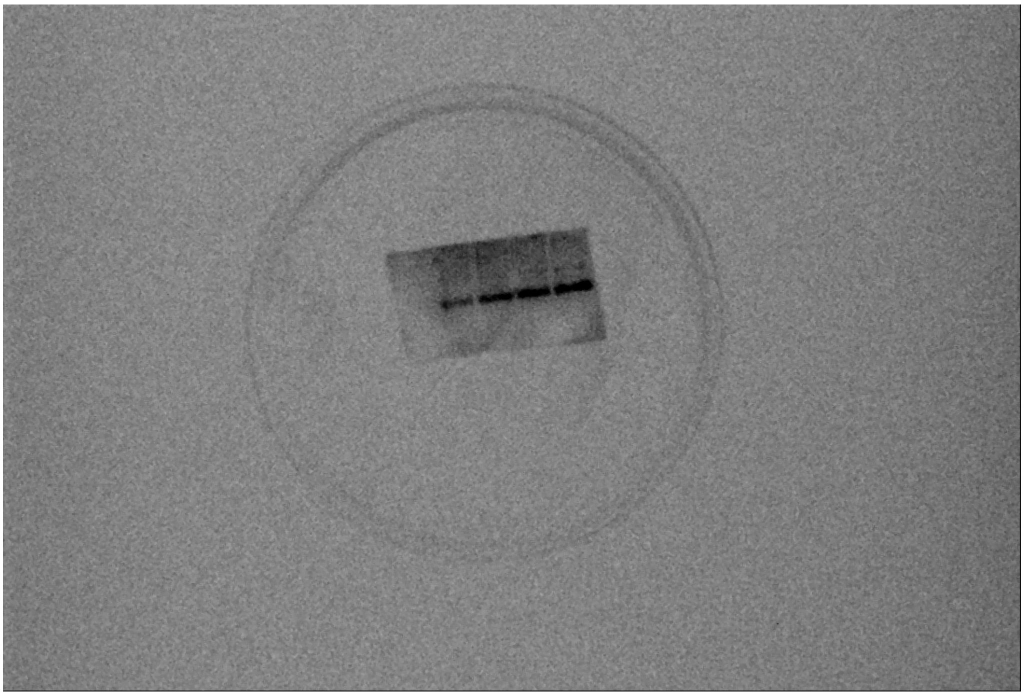

β-actin

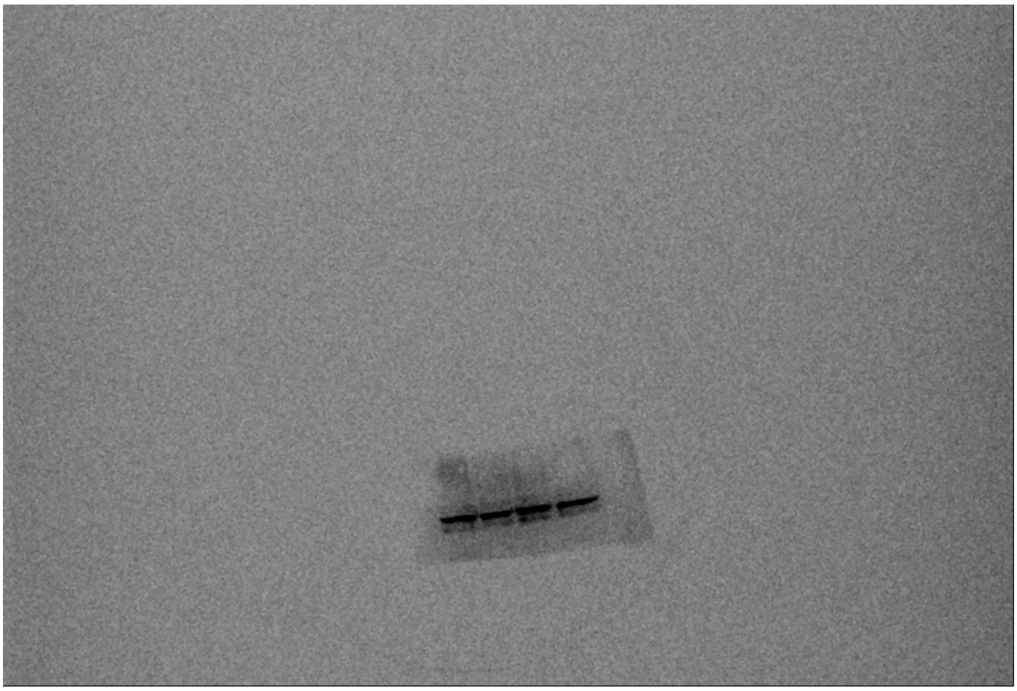

Fig.S4 for Fig.3C

GFAP

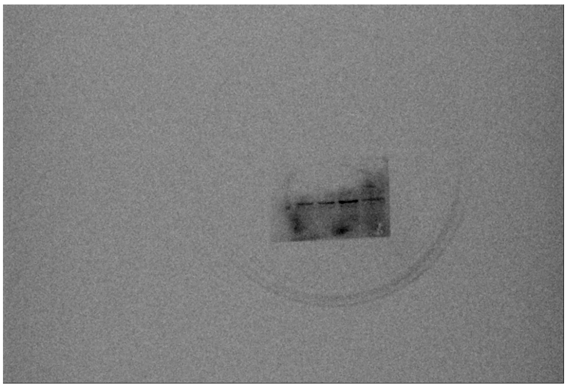

β-actin

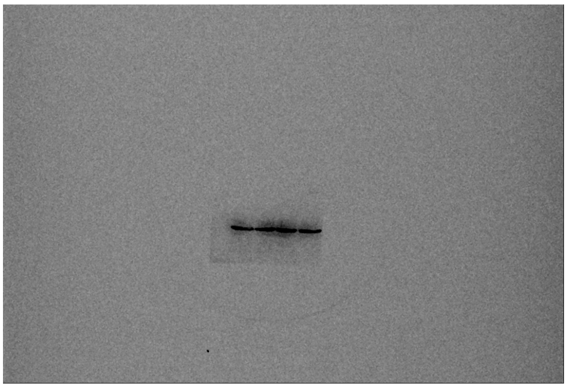

Fig.S5 for Fig.4C

CaMKII

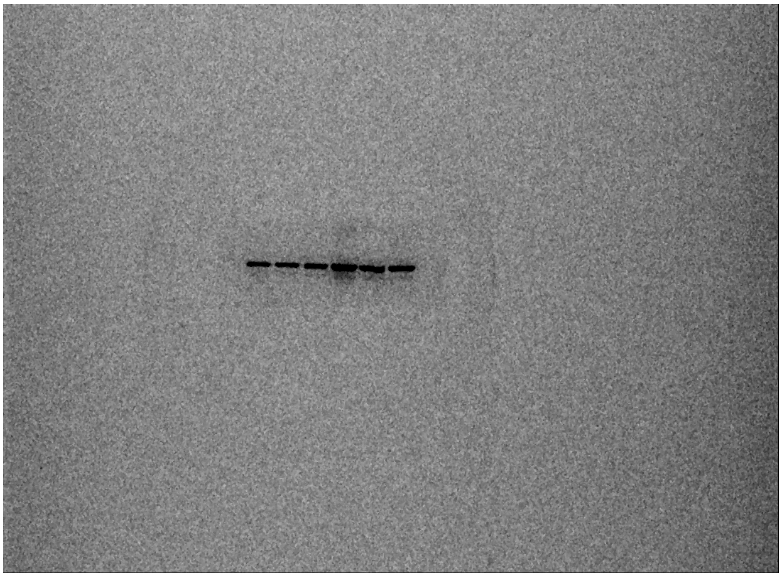

β-actin

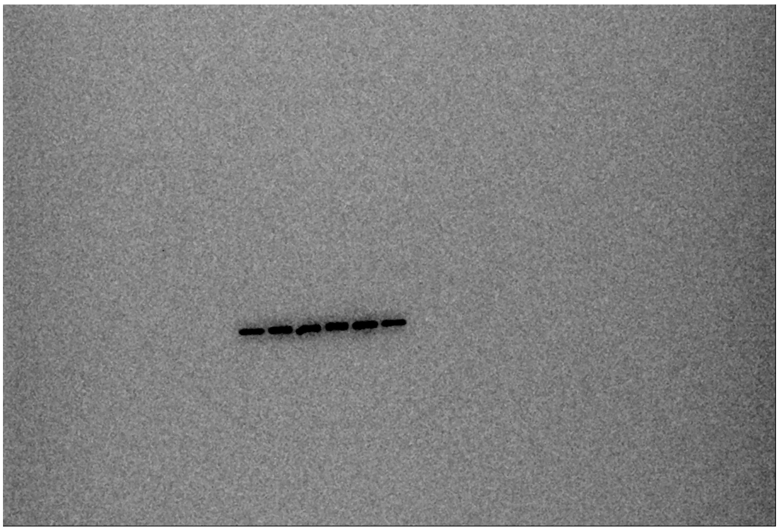

Supplement: Supplementary file 1 [file toxics-14-00595-s001.zip › toxics-4352740-supplementary.pdf]
